# Editorial: A year in review: discussions in pulmonary medicine

**DOI:** 10.3389/fmed.2024.1434562

**Published:** 2024-05-31

**Authors:** Yuan Wang, Mehdi S. Mirsaeidi, Chunxue Bai, Dawei Yang

**Affiliations:** ^1^Department of Pulmonary and Intensive Care Medicine, Zhongshan Hospital, Fudan University, Shanghai, China; ^2^Division of Pulmonary, Critical Care and Sleep, College of Medicine-Jacksonville, University of Florida, Gainesville, FL, United States; ^3^Shanghai Engineer and Technology Research, Center of Internet of Things for Respiratory Medicine, Shanghai, China; ^4^Shanghai Key Laboratory of Lung Inflammation and Injury, Shanghai, China; ^5^Shanghai Institute of Infectious Disease and Biosecurity, Shanghai, China; ^6^Shanghai Respiratory Research Institution, Shanghai, China; ^7^Department of Pulmonary and Intensive Care Medicine, Zhongshan Hospital (Xiamen), Fudan University, Xiamen, China

**Keywords:** respiratory disease, COVID-19, critical care, tumor microenvironment, interstitial lung disease

In 2023, respiratory diseases still remain one of the global health concerns. Although the COVID-19 pandemic has eased to some extent, it continues to have a significant impact on the respiratory system, particularly in areas where outbreaks still occur. In addition, other respiratory diseases such as lung cancer, malignant pleural mesothelioma (MPM), pulmonary hypertension and invasive fungal disease continue to be a global health challenge. These diseases cause breathing difficulties, reduced lung function, multi-system involvement, and even life-threatening situations. The medical community continues to work toward improving diagnosis and treatment methods, as well as strengthening prevention measures, including promoting vaccine uptake and health education.

Conventional treatment methods, including medication and surgery treatment, are also applicable to respiratory diseases. Invasive fungal diseases (IFDs), a commonly observed disease among COVID-19 patients, consist of COVID-19-associated pulmonary aspergillosis (CAPA), COVID-19-associated candidiasis (CAC), fusariosis, coccidioidomycosis, mucormycosis, histoplasmosis, saccharomycosis and pneumocystosis (1). A prospective observational single-center study involving 6,335 patients was performed to establish the clinical features of CAPA and CAC through diagnostic tests, the progression of the disease, and the response to treatment. It is important to consider the effect of corticosteroids and immunobiological therapies on the occurrence and fatality of IFD when managing patients with COVID-19. The use of preventive antifungal medications and medications that target IFD should be taken into account as well (Adzic-Vukicevic et al.). Liu et al. presented a systematic review suggesting that minimally invasive surgery could result in satisfactory outcomes, a higher R0 resection rate, and improved short-term and long-term survival compared to open thoracotomy among patients with non-small cell lung cancer (NSCLC).

Specialized therapies for respiratory diseases lie in providing auxiliary and intensive care through devices. In terms of auxiliary care, devices such as oxygen concentrators and ventilators can effectively alleviate and treat symptoms of respiratory and pulmonary diseases. As for intensive care, measures such as artificial ventilation and tracheal intubation are applied to support patients' respiratory function and improve their vital signs. The application of these technologies can effectively save critically ill patients and is an important feature of the respiratory system. Wu et al. conducted one meta-analysis to assess the impacts of various mechanical ventilation modes utilized for critically ill patients, neurally adjusted ventilatory assist turned out to decrease mortality in ICU and proportional assist ventilation might elevate the likelihood of ventilator withdrawal (Wu et al.). One review detailed the alterations in sleep patterns observed in critically ill individuals, explored the connection between the clinical and physiological aspects of critical illness, including post-ICU recovery and sleep, and outline potential strategies put forward to manage disrupted sleep in the intensive care unit (Eschbach and Wang). Another review focuses on the present understanding of pre-capillary pulmonary hypertension caused by interstitial lung disease, with an emphasis on its diagnosis and treatment, recent advancements, and potential developments in the future, Arslan et al. achieved at the conclusion successfully that inhaled Treprostinil could treat this dreadful disease.

As the study on tumor microenvironment deepens, more studies suggest that systemic inflammatory response indicators play an essential role in the prognosis of malignant tumors (2). Particularly, neutrophils, lymphocyte (3), monocyte (4), platelets (5), and platelet-to-lymphocyte ratio (PLR) (6) have been confirmed to affect the prognosis of respiratory system tumor. Some electrolytes, nutritional indicators, and serum tumor markers are also thought to influence the prognosis of malignant tumors (7, 8). Recent studies aimed to identify whether these laboratory variables and markers were significantly correlated with prognosis. Zhang et al. diagnosed 90 patients with MPM, collected all fundamental, clinical, radiographic, and laboratory parameters, and conducted the COX univariable and multivariable analysis. The relationship between the prognosis of patients with MPM and the results of inflammation-related and electrolyte laboratory variables in peripheral blood, such as Calcium, MWR, and PLR, may provide valuable insights for clinicians to assess patient conditions. Lv et al. gathered data on inflammatory and nutritional markers from a total of 5,239 patients who were pathologically diagnosed with NSCLC, divided the patients into four groups: stage I/II operable, stage III operable, stage III inoperable, and stage IV, and found that for patients with stage III and IV NSCLC, some nutritional indicators, serum tumor markers, and inflammatory markers are independent prognostic factors.

This topic contains seven valuable articles for the characteristics, therapies and prognostic evaluation of respiratory system diseases. Additional articles are needed to offer more compelling evidence to enhance our understanding of these diseases.

## Author contributions

YW: Formal analysis, Investigation, Resources, Writing – original draft. MM: Project administration, Resources, Supervision, Validation, Writing – review & editing. CB: Conceptualization, Funding acquisition, Project administration, Resources, Supervision, Validation, Writing – review & editing. DY: Conceptualization, Formal analysis, Funding acquisition, Investigation, Methodology, Project administration, Resources, Supervision, Validation, Visualization, Writing – original draft, Writing – review & editing.
